# Emoji Use in the Electronic Health Record

**DOI:** 10.1001/jamanetworkopen.2025.53770

**Published:** 2026-01-14

**Authors:** David A. Hanauer, Gavin C. Raab, Shira N. Hanauer, Lisa Ferguson, Kellen McClain, Guan Wang, Michelle Rozwadowski, Sung W. Choi

**Affiliations:** 1 Department of Learning Health Sciences, University of Michigan Medical School, Ann Arbor; 2 Department of Pediatrics, University of Michigan Medical School, Ann Arbor; 3 College of Arts and Sciences, Cornell University, Ithaca, New York

## Abstract

This cross-sectional study investigates rates and characteristics of emoji use in clinical notes within electronic health records.

## Introduction

Emojis are small digital images that visually express emotions, ideas, or concepts. Their use has been reported in health care settings, such as texting between clinicians,^1^ but we are unaware of studies characterizing emojis within electronic health record (EHR) clinical notes, including patient portal messages.

## Methods

This cross-sectional study was approved by the University of Michigan Institutional Review Board with a waiver of informed consent because it was deemed minimal risk, without direct involvement of human participants. The STROBE reporting guideline was followed. Our dataset included 218.1 million notes from 1.6 million patients created between January 1, 2020, and September 30, 2025, at Michigan Medicine. All emojis in notes were identified. Details, including date, note type, and patient age, were recorded. We randomly selected 100 emoji-containing notes each from 2024 and 2025 and qualitatively coded emoji use along 5 dimensions. Two independent raters (G.C.R. and S.N.H.) performed the coding, with interrater reliability assessed via Cohen κ. A third reviewer (D.A.H.) resolved discrepancies between raters and finalized the dataset. Additional details are in the eMethods in Supplement 1.

## Results

There were 372 distinct emojis used within 4162 notes (Figure). Approximately one-quarter (1011 notes [24.3%]) contained more than 1 emoji (maximum = 32; median = 4). Emoji usage rates remained mostly stable at 1.4 notes with emojis per 100 000 notes from 2020 to 2024, increasing to 10.7 per 100 000 notes by quarter 3 of 2025.

**Figure.**
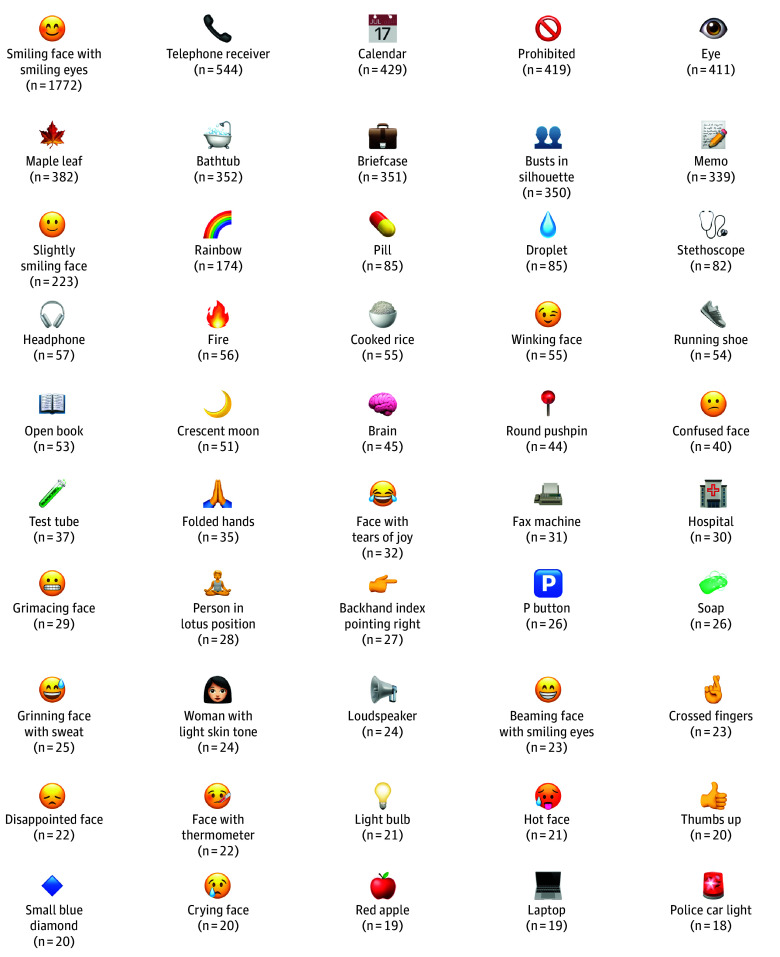
Fifty Most Common Emojis The 50 most frequently occurring emojis found in electronic health record notes, including their official names and the number of notes containing the emoji, are presented. Emojis are shown using the open-source Noto Color Emoji font due to copyright restrictions on other versions.

Among notes containing emojis, the distribution by Unicode category was smileys and emotion (2434 notes [58.5%]), objects (881 notes [21.2%]), people and body (734 notes [17.6%]), symbols (496 notes [11.9%]), animals and nature (443 notes [10.6%]), travel and places (371 notes [8.9%]), food and drink (140 notes [3.4%]), activities (27 notes [0.6%]), flags (4 notes [<0.1%]), and component (3 notes [<0.1%]). Portal messages to patients were the most common EHR notes containing emojis (1477 notes [35.5%]), followed by telephone encounters (1185 notes [28.5%]), encounter summaries (637 notes [15.3%]), progress notes (579 notes [13.9%]), patient instructions (265 notes [6.4%]), and 7 other types (19 notes [0.5%]). Because our Epic patient portal restricts patients from including emojis, none were in the 34.5 million patient portal messages.

The rate of portal messages to patients containing emojis per 100 000 portal messages stratified by decade of patient age was 1.0 (0-9 years), 3.8 (10-19), 2.8 (20-29), 2.5 (30-39), 2.4 (40-49), 1.9 (50-59), 2.2 (60-69), 3.3 (70-79), 2.6 (80-89), and 1.3 (90-99) messages. The Table summarizes the coding of emoji use across 200 clinical notes, detailing the distribution of categories, such as originator, recipient, uniqueness, emotional content, and usage type. Among 21 emojis generated by patients and family members, 19 were added to the EHR through a copy-pasted email, 1 was from a copied text message, and 1 was entered via a questionnaire uploaded to the portal.

**Table.** Distribution of Emoji Coding by Dimension and Category

Dimension	Notes, No. (%) (N = 200)	Cohen κ^a^
Emoji originator: Who introduced the emoji into the note?		
Patient or family	21 (10.5)	0.95
Clinical team, not patient or family^b^	178 (89.0)	
Unable to determine	1 (0.5)	
Intended recipient: Who is the primary audience or presumed recipient for the emoji?		
Patient or family	128 (64.0)	0.91
Clinical team, not patient or family^b^	70 (35.0)	
Unable to determine	2 (1.0)	
Uniqueness: Does the emoji appear to be uniquely added or part of a reusable template?		
Uniquely added	117 (58.5)	0.93
Templated	82 (41.0)	
Unable to determine	1 (0.5)	
Emotional content: Does the selected emoji represent an emotion or feeling (vs an informational or symbolic use)?		
Emotion or feeling	119 (59.5)	0.93
Informational or symbolic use	81 (40.5)	
Unable to determine	0	
Usage: How does the emoji function within the note?		
Replacing (eg, take 💊 as prescribed)	2 (1.0)	0.90
Augmenting (eg, eat more carrots **🥕**)	83 (41.5)	
Stand-alone (eg, have a wonderful day 🌈)^c^	114 (57.0)	
Unable to determine	1 (0.5)	

^a^ Between 2 independent reviewers, prior to adjudication for the finalized dataset.

^b^ This could include people such as school counselors, employees at health-related nonprofit organizations, and others.

^c^ Not directly connected to any specific word or concept.

## Discussion

Although emoji use in the EHR was rare overall in this cross-sectional study, we identified substantially more distinct emojis than previously reported in clinician-to-clinician texting (372 vs 42 emojis).^1^ We have no explanation for why emoji use increased recently given that there have been no changes to our EHR to enable or encourage their use.

Emojis in clinical documentation, particularly within patient-directed communications, raise questions about potential misinterpretation. Emoji interpretations may differ by age group,^2^ and notable concerns exist regarding their use among older adults, although findings are mixed.^3,4^ We found that emojis were sent in portal messages to patients aged 70 to 79 years at the second highest rate, after those aged 10 to 19 years.

Institutions may consider tracking emoji use and develop guidelines for clinical documentation and messaging. Study limitations include single-center design, inability to link emojis to clinical outcomes, and no data on clinician or patient interpretability of emojis. Future research should address these gaps and explore the association of emojis with clinical communication.

## Notes

Supplement 1.
eMethods.

Supplement 2.
Data Sharing Statement
