# Supplementary material for: Emoji Use in the Electronic Health Record
Source: JAMA Netw Open. 2026 Jan 14;9(1):e2553770. doi: 10.1001/jamanetworkopen.2025.53770 (PMC12805451; doi:10.1001/jamanetworkopen.2025.53770)
Supplement: Supplement 1. — eMethods. [file jamanetwopen-e2553770-s001.pdf]

Supplemental Online Content

Hanauer DA, Raab GC, Hanauer SN, et al. Emoji use in the electronic health record. *JAMA Netw Open*. 2026;9(1):e2553770. doi:10.1001/jamanetworkopen.2025.53770

eMethods.

This supplemental material has been provided by the authors to give readers additional information about their work.

© 2026 Hanauer DA et al. *JAMA Network Open*.

eMethods.

Study Approval

This study was reviewed and approved by the University of Michigan Institutional Review Board. A waiver of informed consent was provided for this study involving the secondary use of data.

Study name: An evaluation of emojis in the electronic health record

IRB number: HUM00281440

Initial approval date: September 24, 2025

Data Source

The source of data for this study included clinical electronic health record (EHR) notes stored in the University of Michigan (UM) Electronic Medical Record Search Engine (EMERSE). EMERSE allows for rapid searching across all notes, so that notes containing emojis can be easily identified. The notes within EMERSE at UM are primarily sourced from our Epic instance. Unlike other institutions that use EMERSE, we obtain most of our Epic notes at the time they are created through an outbound HL7 feed that preserves the note in its original rich text format (RTF). Our indexing process for EMERSE converts the RTF to HTML and preserves Unicode characters such as emojis.

Note that for many institutions with Epic, clinical notes are obtained through the reporting database Clarity, where all formatting is stripped to store plain text documents. Based on our local Clarity resource, emojis are not retained in the standard Clarity notes and are instead replaced by '??'. As a result, institutions with Epic seeking to identify emojis within their notes will have to verify if any data loss might have occurred when moving the data to Clarity.

Our patient portal messages are stored in Epic Clarity, but unlike most other notes in Clarity, these are stored with their original formatting preserved, including emojis. For these portal notes, we obtain them directly from Clarity for indexing with EMERSE.

© 2026 Hanauer DA et al. *JAMA Network Open*.

Emoji resource

The emojis we searched for came from a comprehensive Unicode Consortium-generated list of 5,225 symbols, which includes official emoji names and categories (https://unicode.org/Public/emoji/latest/emoji-test.txt), accessed on October 3, 2025.

Qualitative Coding

To better understand how emojis are used in clinical notes, we iteratively developed a codebook comprising five dimensions for evaluating emojis within the notes.

Once an initial codebook was developed, two reviewers familiar with emoji usage (ages 18 and 22 at the time of the study) independently coded an initial set of 10 emoji-containing notes. Coding challenges were discussed within the team, and the codebook was subsequently refined. The finalized codebook is included at the end of this document.

For the main analysis, we randomly selected 200 clinical notes containing emojis (100 notes from 2024 and 100 from 2025. The same two reviewers then independently applied the revised codebook to these 100 notes.

Inter-rater agreement between the two reviewers was assessed with Cohen's kappa using the **irr** package in R version 4.5.2.

A third reviewer (DAH) adjudicated all coding discrepancies and determined the final coding for the dataset reported here.

Because it was unclear how a patient or family member could generate an emoji in an EHR note (since the portal forbids emoji entry) we conducted an additional review of notes where the emoji was determined to originate from a patient or family member. For these notes the two reviewers recorded the likely source from which the emoji originated, which included “email”, “text message”, “other (write-in)”, or “unable to determine”). Any discrepancies were adjudicated by the third reviewer.

Data availability

A de-identified dataset of all emojis identified in clinical notes, including metadata (note type, quarter, and patient age), is available upon request. The final-coded dataset is also available. Original clinical notes cannot be shared due to protected health information.

© 2026 Hanauer DA et al. *JAMA Network Open*.

Codebook for Emoji Coding in Clinical Notes/Portal Messages

Version 1.2
2025-11-07

General Instructions

1. **Scope:**
   - Only notes containing emojis will be provided for the coding.
2. **Multiple Emojis:**
   - If a message contains more than one emoji, code **only the first emoji that appears in the text**. This reduces coder bias and maintains consistency across coders.
3. **Coding Approach:**
   - Use **note/message context** and your judgment to make determinations. Read surrounding text, sender information, and any relevant note structure to guide your decision.
4. **Importan**t:
   - The notes you will review contain protected health information (PHI) so they must be kept secure and not shared. Do not move them or copy them to any other location.

© 2026 Hanauer DA et al. *JAMA Network Open*.

Dimension 1: Emoji Originator

Definition: Identify who introduced the emoji into the message.

- **0 = Patient/Family Member:**
  The emoji was first included by the patient or their family member (e.g., an email, portal message, or section of note attributed directly to a patient or caregiver).
  - *Example:* Note states, “Patient messaged: ‘I am feeling much better 😊’.”
  - *Example:* Chart includes, “From patient portal: ‘I’m worried 😟’.”
- **1 = Clinician/Non-Patient/Non-Family Member:**
  The emoji was first added by a healthcare professional (physician, nurse, admin, etc.), not the patient or family member.
  - *Example:* Physician writes, “Your blood pressure is improving 👍.”
  - *Example:* Nurse message: “Let us know if you need help 🆘.”
- **2 = Unsure:**
  If context does not make it clear who introduced the emoji, or if the message is ambiguous (e.g., “copied below” without specification), use this code.
  - *Example:* Note lacks attribution, message simply says, “Feeling okay 🙂,” with no sender or recipient details.

Tip: When unsure, add a brief comment describing the ambiguity.

© 2026 Hanauer DA et al. *JAMA Network Open*.

Dimension 2: Intended Recipient

Definition: Identify the primary audience or presumed recipient for the emoji or the message.

- **0 = Patient/Family Member:**
  Message or note is directed to the patient or their family/caregiver.
  - *Example:* Message directly addresses patient or family: “Take care until we see you next 🌞.”
  - *Example:* Discharge instructions: “Call our office if you need help 🆘.”
- **1 = Another Clinician/Care Team Member/Other Non-Patient, Non-Family Member:**
  The emoji is directed at, or the message is intended for, another healthcare provider, such as a nurse, physician, pharmacist, or other team member.
  - *Example:* “MD Progress Note: ‘ECHO looks promising 👍’.”
  - *Example:* Handoff note: “Patient stable overnight 💤.”
- **2 = Unsure:**
  Recipient cannot be clearly identified based on message context.

Tip: If message is shared with multiple audiences, select recipient most directly addressed. If completely ambiguous, select “Unsure” and add a note.

© 2026 Hanauer DA et al. *JAMA Network Open*.

Dimension 3: Uniqueness (Templated vs. Unique Addition)

Definition: Determine if the emoji appears to be part of a reusable template or was uniquely added.

- **0 = Uniquely Added:**
  The emoji appears to be specifically inserted for the message; it is not part of a prewritten template.
  - *Example:* Free-text from a nurse: "Have a great weekend 🎉."
  - *Example:* Doctor writes: "Please follow up if your symptoms get worse 😟."
- **1 = Templated:**
  The emoji is likely part of a standard message or set of reusable instructions.
  - *Example:* Note includes, "Take medication as directed 💊," and this phrase is known or suspected to appear in multiple patient instructions.
  - *Example:* Message format matches a documented template in use, including the emoji.
- **2 = Unsure:**
  The coder cannot clearly determine if the emoji is templated or uniquely added (e.g., message style is generic, but template presence is unclear).

Tip: Can add a comment about why a decision was mode for any of the determinations.

© 2026 Hanauer DA et al. *JAMA Network Open*.

Dimension 4: Emotional Content

Definition: Does the selected emoji represent an emotion or feeling (vs. a purely informational or symbolic use)?

- **0 = No:**
  Emoji does **not** represent emotion; it provides information, clarification, or serves as a non-emotional icon.
  - *Example:* “Take one tablet 💊” (pill icon is informational, not emotional).
  - *Example:* “Eat more carrots 🥕” (carrot = food item).
- **1 = Yes:**
  Emoji is intended to convey an emotion (e.g. sadness, happiness, excitement, reassurance, worry).
  - *Example:* “We’re sorry for your loss 😢.”
  - *Example:* “Glad you’re feeling better 😊.”
- **2 = Unsure:**
  Cannot determine if the emoji represents emotion, or if context is ambiguous.
  - *Example:* “You’re done 🍾” (champagne could be celebratory or just a congratulatory icon).

Tip: Can refer to Emoji Meanings List for ambiguous cases, but use context primarily.

© 2026 Hanauer DA et al. *JAMA Network Open*.

Dimension 5: Usage

Definition: Describe how the emoji functions within the message.

- **0 = Replacing a Word/Concept:**
  The emoji stands **in place of** a word or an idea that could be stated textually.
  - *Example:* “If you feel 😢 call us.” (Sad face replaces the concept of “sad”).
  - *Example:* “Take 💊 as prescribed” (pill replaces “medicine”).
- **1 = Augmenting a Word/Concept:**
  Emoji is **added alongside** a word or idea to reinforce or supplement meaning.
  - *Example:* “Eat more carrots 🥕.” (carrot added after the word “carrots”).
  - *Example:* “Congratulations! 🎉” (“🎉” emphasizes celebration).
- **2 = Standalone Use (Not Directly Connected to Any Specific Word/Concept):**
  Emoji is **used independently**, not tied to a specific word, often in greetings, signatures, or as an overall message tone.
  - *Example:* “Have a wonderful day 🌈.”
  - *Example:* Message ends with “😊” after a closing signature.
- **3 = Unsure:**
  Cannot determine clearly if emoji is replacing, augmenting, or standalone.

Tip: If ambiguous, pick “Unsure” and note why.

© 2026 Hanauer DA et al. *JAMA Network Open*.
