# Supplementary material for: Emoji Use in the Electronic Health Record
Source: JAMA Netw Open. 2026 Jan 14;9(1):e2553770. doi: 10.1001/jamanetworkopen.2025.53770 (PMC12805451; doi:10.1001/jamanetworkopen.2025.53770)
Supplement: Supplement 2. — Data Sharing Statement [file jamanetwopen-e2553770-s002.pdf]

Data Sharing Statement

Hanauer. Emoji Use in the Electronic Health Record. *JAMA Netw Open*. Published online January 14, 2026. doi:10.1001/jamanetworkopen.2025.53770

Data
Data available: Yes
Data types: Deidentified participant data, and coded data from the notes
How to access data: Data are available upon request
When available: With publication

Supporting Documents
Document types: Other (please specify)
Additional Information: We include a supplemental file with additional methodological details, including the codebook we used for the qualitative coding component of the study.
How to access documents: Available as a supplemental file with the publication.
When available: With publication

Additional Information
Who can access the data: Data will be made available to anyone who requests it.
Types of analyses: Data could be used for any purposes other than trying to re-identify individuals.
Mechanisms of data availability: Data will be made available at no cost. No support is required; no data access agreement is required.
